# RNA Sequencing Reveals LINC00167 as a Potential Diagnosis Biomarker for Primary Osteoarthritis: A Multi-Stage Study

**DOI:** 10.3389/fgene.2020.539489

**Published:** 2021-01-14

**Authors:** Liying Jiang, Yiqin Zhou, Junjie Shen, Yi Chen, Ziyuan Ma, Yuhui Yu, Minjie Chu, Qirong Qian, Xun Zhuang, Shengli Xia

**Affiliations:** ^1^Shanghai Key Laboratory for Molecular Imaging, Shanghai University of Medicine & Health Sciences, Shanghai, China; ^2^Department of Joint Surgery and Sports Medicine, Shanghai Changzheng Hospital, Second Military Medical University, Huangpu, China; ^3^Department of Epidemiology, School of Public Health, Nantong University, Nantong, China; ^4^Department of Orthopedics, School of Medical, Strategically Strategic Medical University, Guiyang, China; ^5^Department of Orthopedics, Shanghai University of Medicine & Health Sciences Affiliated Zhoupu Hospital, Shanghai, China

**Keywords:** osteoarthritis, RNA-seq, marker, peripheral blood leukocytes, lncRNA

## Abstract

**Objectives:**

Given the roles played by lncRNA in human diseases and the high incidence of OA, this study investigated the pivotal pathways involved in the disease and identified potential biomarkers for OA diagnosis.

**Methods:**

We first performed an exploration of RNA-sequencing in peripheral blood leukocytes from six subjects (3 OA and 3 healthy controls). Promising candidate lncRNAs were evaluated in first stage validation using a GEO dataset (GSE114007) of 38 subjects (20 OA and 18 healthy controls), followed by a second stage validation using quantitative PCR analysis with 101 subjects (67 OA and 34 controls). The third stage investigated the potential value of validated lncRNA in the early diagnosis of OA in peripheral blood leukocytes from a total of 120 participants (60 cases and 60 controls).

**Results:**

The dataset identified a total of 1,380 up-regulated and 719 down-regulated mRNAs and 5,743 up-regulated and 7,384 down-regulated lncRNAs. The up-regulated DEGs were mainly enriched in the extracellular matrix, while the down-regulated DEGs were mainly enriched in the IL-17 and wnt signaling pathways. 18 overlapping candidate lncRNAs survived after first-stage validation. 3 hub lncRNAs were selected for the second validation stage and qualified in an external sample, and lncRNA *LINC00167* was further confirmed with a similar result (down-expressed in both stages). Receiver operating characteristic analysis showed that *LINC00167* can distinguish OA cases from healthy controls with a high area under the curve of 0.879 (95%CI: 0.819, 0.938; *P* < 0.001), with a sensitivity of 80.7% and specificity of 83.5%.

**Conclusion:**

The expression profile of OA was identified and critical pathways were elucidated by an integrated approach to RNA-seq from easily accessible blood. *LINC00167* may serve as a potential early diagnosis marker for OA in clinical practice. The detailed mechanism of action of this lncRNA requires further elucidation in future studies.

## Introduction

Osteoarthritis (OA) is a common and disabling condition characterized by articular cartilage degradation. It creates substantial and increasing burdens for healthcare with notable implications for elderly individuals (Hermann et al., 2018; Piwowar et al., 2018). The etiology of OA is not fully understood, however, comprehensive studies have suggested that OA is a result of complex interactions between environmental factors (including lifestyle factors) and genetics, whose development is primarily a complicated process covering broad mechanisms and a number of molecules (Jayasuriya et al., 2018; Hunt et al., 2019; Maly et al., 2019). Given the irreversibility of its progress, early prevention and precisive diagnosis of OA is of utmost importance (Kijowski et al., 2019; Kloppenburg and Berenbaum, 2020).

Long non-coding RNAs (lncRNAs), which are conventionally defined as being over 200 nucleotides with an absence of coding ability, are emerging as key regulators of gene expression (Jarroux et al., 2017). Several studies have shown that lncRNAs play important roles in malignancy and chronic diseases, including OA (Peffers et al., 2018; Razmara et al., 2019; Wu et al., 2019). In a study by Fu et al. (2015) there were 3,007 up-regulated lncRNAs and 1,707 down-regulated lncRNAs in knee OA cartilage compared to normal samples and some of them were predicted to have target genes that were associated with OA. Numerous research has shown that lncRNAs play a critical role in the regulation of chondrocyte and synovial cell survival (Dou et al., 2017; Zheng et al., 2017; Cao et al., 2018), as well as in mediating inflammation (Pearson and Jones, 2016) and angiogenesis (Cen et al., 2017). A recent study reported that NON-HSAG034351 was the hub lncRNA down-regulated in synovial tissue and could play a central role in the pathological progression of OA (Shui et al., 2020).

Large-scale exploration has been conducted by comparing OA cases and healthy controls based on blood samples (Ramos et al., 2014). Compelling studies have confirmed that some specific lncRNAs such as lncRNA-ATB, lncRNA MIR4435-2HG, and lncRNA DILC dysregulated in OA blood samples and regulated chondrocyte cell proliferation and apoptosis (Dang et al., 2018; Huang et al., 2019; Xiao et al., 2019). These studies have demonstrated the importance of lncRNAs in OA blood. However, the profile of lncRNAs and their potential biological functions in blood samples have not been systematically characterized in OA patients (van Spil and Szilagyi, 2019). Importantly, a study has reported that the expression of exosomal lncRNAs in blood showed no significant difference between OA and non-OA, while for synovial fluid samples, the expression of exosomes in early OA and late-stage OA was markedly higher than that in controls (Zhao and Xu, 2018). Considering the tissue specificity of lncRNAs in OA, we explored hub lncRNAs that are abnormally expressed in the synovial tissues and cartilage, and blood.

This study sequenced a complete transcriptome RNA in peripheral blood leukocytes (PBL) of OA and controls (3 cases and 3 controls). A stepwise screening approach was also applied to identify potential hub lncRNAs. It then validated the expression levels of potential hub lncRNAs (67 cases and 34 controls). A further diagnostic test was conducted to investigate the potential ability to OA diagnosis for validated lncRNAs (60 cases and 60 controls). The study aimed to investigate the pivotal pathways involved in OA pathophysiology and to identify potential biomarkers for OA diagnosis in PBL, which could provide a roadmap to pinpoint candidates for future lncRNA-based diagnostics and therapies.

## Materials and Methods

### Sample Collection

This study involved three stages. In the RNA-seq screening stage, three OA cases over 40 years old were recruited from the department of orthopedics of Shanghai Zhoupu hospital in March 2018. Three healthy controls were randomly selected from a pool of more than 100 individuals who participated in routine health surveillance in Zhoupu hospital and matched with OA cases according to age and gender. Methods of diagnosing OA have been described in our previous study (Chu et al., 2017). During the second validation stage, 67 OA patients (knee OA: 54; other OA: 13) and 34 OA-free controls were recruited from the Department of Joint Surgery and Sports Medicine in Shanghai Changzheng hospital, between April and December 2019. In the third stage, another 60 OA cases were recruited from the same hospital from April to June 2020 to investigate the potential value of the validated lncRNA in OA diagnosis. Finally, 60 healthy controls were randomly selected from a pool of more than 500 individuals, who participated in routine health surveillance at the same hospital in June 2020. The healthy control subjects were recruited from a survey and had no signs or symptoms of arthritis or joint diseases. Secondary OA patients such as those with inflammatory arthritis, rheumatoid, bone fracture, and developmental dysplasia were excluded.

All participants were Han Chinese, aged 40 over, and were not related to one another. Each subject completed an interview by a trained investigator using a structured questionnaire to collect information regarding demographic characteristics as well as environmental exposure, including recreational sports activities, previous knee injuries, family history of OA and other diseases, and clinical manifestations of OA.

The study was reviewed and approved by the Ethics Committee of Shanghai University of Medicine and Health Sciences. All participants signed written informed consent.

### RNA-Sequencing

Five milliliter whole blood was kept at room temperatures for 2 h, followed by centrifugation at 1,500 g for 20 min to obtain white blood cells. A lymphocyte isolation kit was used to purify lymphocytes. Total RNAs were extracted from the PBL of three cases and three healthy controls using TRIzol (Invitrogen, Carlsbad, CA, United States).

The RNA samples were sent to Gminix Biotechnology Co., Ltd. (Shanghai, China) for RNA-seq, only samples with high quality RNA (RNA Integrity Number ≥ 8.0) were used in the subsequent construction of RNA-seq libraries. Sequencing was performed on an Illumina HiSeq 2,500 sequencing platform with 10 M reads (Illumina, San Diego, CA, United States). Hisat2 (Langmead, 2010) was used to compare the differently expressed mRNAs and lncRNAs and featureCounts (Liao et al., 2014) were adopted to annotate and quantify mRNAs and lncRNAs. A principal component analysis (PCA) of normalized data was performed in the present study using R software. PCA was used for unsupervised multivariate analyses to determine the directions of maximum covariance without referring to class labels (OA/Normal). Then, counts in the two groups were analyzed by DESeq2 (Love et al., 2014), and differently expressed genes (DEGs) were screened. mRNAs and lncRNAs with an absolute value fold changes (FC) ≥ 2 and *P* < 0.05 were considered as significantly differently expressed.

### Enrichment Analysis for DEGs

GO is a tool for the unification of biology, which collects structured, defined, and controlled vocabulary for large scale of gene annotation, including biological processes (BP), cellular component (CC), and molecular function (MF) (Kramarz and Lovering, 2019). KEGG database classifies correlating gene sets into their respective pathways. We undertook enrichment analyses for DEGs of the intersection genes across two datasets to explore the potential roles of co-differently DEGs and hub lncRNAs in the current analysis (Du et al., 2014). *P* < 0.05 for GO enrichment and *P* < 0.1 and count > 2 for the KEGG pathway were considered for further analysis. The Database for Annotation, Visualization, and Integrated Discovery (DAVID) (Huang et al., 2007), a comprehensive set of functional annotation tools, was employed to relate the functional terms with gene lists by a clustering algorithm.

### First-Stage Validation Based on GEO Dataset

The microarray datasets GSE114007 of articular cartilage were downloaded from the Gene Expression Omnibus (GEO) database^1^, which used GPL11154 Illumina HiSeq 2,000 and GPL18573 Illumina NextSeq 500 as platforms, to identify differential expression (Illumina, Inc.). RNA-seq was performed on 18 normal and 20 OA human knee cartilage tissues. Subsequently, the gene expression data were subjected to identical processing using the Robust Multichip Average function within the limma R package (version 3.24.15). The criteria were as follows: mRNAs and lncRNAs with FC #x2265; 2, as well as *P* < 0.05 were considered to be differently expressed. The candidate lncRNAs of co-expression pairs with an absolute value of the Pearson correlation coefficient of #x2265; |0.995| were selected as hub lncRNAs.

### Second-Stage Validation Using qRT-PCR

To validate the expression level among OA cases and the healthy control group, 101 samples (67 cases and 34 controls) were collected and used for quantification, and the relative expression level in each group was performed by quantitative reverse transcription polymerase chain reaction (qRT-PCR) (Applied Biosystems, Foster City, CA). After the Pearson correlation coefficient screening, we randomly selected 3 hub lncRNAs from candidate lncRNAs to perform validation using qRT-PCR, including the metastasis-associated lung adenocarcinoma transcript 1 (*MALAT1)*, long intergenic non-protein coding RNA167 (*LINC00167*), and the HLA complex group 9 (*HCG9*). The result for *HCG9* was unavailable because of test failure. The primer designs are as follows:

*LINC00167*-142-F: GGTGGCACTGGACTTAGATGAG;

*LINC00167*-142-R: AAAGGGAGCATTCAAGGGACT;

*MALAT1*-256-F: AGTCCAGGAGCCAGTGCG;

*MALAT1*-256-R: TGCCGACCTCACGGATTT;

Total RNA was reverse-transcribed to cDNA using Super-ScriptTM III Reverse Transcriptase (Invitrogen). The qRT-PCR was performed using 2 × SYBR Green PCR Master Mix (Arraystar) on an Applied BiosystemsViiA 7 Real-time PCR System. The final reaction volume was 10 μL and contained 5 μL of SYBR Green PCR Master Mix (2 ×), 0.5 μL of the forward and reverse primers (10 μM), 2 μL of cDNA, and 2 μL of double-distilled water. The qRT-PCR reaction conditions were as follows: denaturation at 95°C for 10 min, followed by 40 cycles of 95°C for 10 s and 60°C for 60 s. GAPDH was used as internal control. Analysis of relative expression was calculated using the 2^–ΔΔ*Ct*^ method (Ct, the threshold cycle to detect fluorescence) (Fu et al., 2015). All reactions were run in triplicate.

### Third-Stage Validation for the Hub lncRNAs

An independent sample was conducted based on the hub lncRNAs. Models were evaluated by a receiver operating characteristic (ROC) analysis using the relative expression for hub lncRNAs and an area under the curve (AUC) criterion.

### Statistical Analysis

Body mass index (BMI) was calculated by dividing the weight (kg) by the squared height (m^2^), and the participants were divided into four categories according to criteria recommended by the Working Group on Obesity in China (Underweight: BMI < 18.5 kg/m^2^; Normal: 18.5 ≤ BMI < 24; Overweight: 24 ≤ BMI < 28; and General obesity: BMI #x2265; 28) (Zhou and Cooperative Meta-Analysis Group of the Working Group on Obesity in China., 2002). lncRNAs with an absolute value fold changes #x2265; 2 and *P* < 0.05 were considered as significantly differently expressed. For GO and pathway analysis, Fisher’s exact test was employed to evaluate the significance of GO terms and Pathway identifiers enrichment in the differentially expressed genes. Pearson correlation coefficient was utilized to test the correlation between co-expressed DEG-lncRNA pairs. The qRT-PCR datasets were presented as a scatter diagram with the means. Analysis of variance (ANOVA) was used to evaluate the expression differences of specific lncRNAs. The qualitative data were tested by the chi-square test. A value of ***P*** < 0.05 was considered statistically significant. All analyses were performed with SPSS 22.0 (IBM Corp) and GraphPad Prism version 8.0 (GraphPad Software, lnc.).

## Results

**FIGURE 1:**
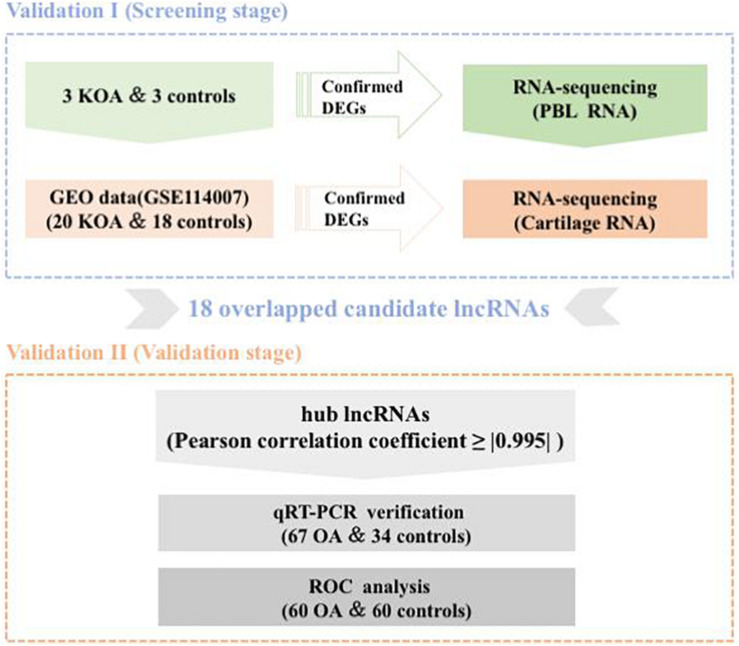
Schematic of study design.

A schematic diagram of the study design is displayed in Figure 1. To estimate false-positives, the procedure was performed using randomized labels for the sample. The assessment results were compared with the original classification based on PCA. PCA revealed obvious differences between the normal controls and OA samples. With an adjusted *P*< 0.05 and | log2FC| > 1 as the cutoff criteria, a total of 1,380 up-regulated and 719 down-regulated mRNAs were identified in our sequencing dataset, together with 5,743 up-regulated and 7,384 down-regulated lncRNAs. After the screening of sequencing samples and the GEO database, the two comparison datasets shared 18 overlapping candidate lncRNAs (Table 1). The characteristics of the screening RNA-seq stage are shown in Table 2.

**TABLE 1:** 18 overlapping RNAs based on internal RNA-seq samples and GSE114007 dataset.

**Gene symbol**	**log 2FC**	***P*-value**
*FLJ13224*	−1.739840836	0.000247
*LINC00272*	2.936771991	0.004111
*HCG9*	6.363270277	0.009919
*PROSER2-AS1*	−5.659371754	0.011373
*SLC2A1-AS1*	1.263846113	0.049066
*HM13-AS1*	−1.875272331	0.042688
*LINC00309*	−2.983855636	0.010667
*MORF4L2-AS1*	2.541198599	8.87E-07
*RGS5*	1.939768295	1.95E-07
*LINC00167*	-1.797369904	1.16E-10
*A2M-AS1*	1.908460663	6.27E-07
*LINC00535*	6.084917886	0.002716
*FAM13A-AS1*	1.006015734	0.009808
*MALAT1*	2.772674305	2.72E-12
*RHPN1-AS1*	−2.11356017	9.64E-05
*ADPGK-AS1*	−1.612490316	3.19E-10
*LINC00622*	2.005605556	0.003978
*CDR1*	2.857618505	6.34E-16

*FC, fold change.*

**TABLE 2:** Characteristics of the subjects enrolled for lncRNA expression analysis in the study.

**Variables**	**Screening (RNA-seq)**			**Second-stage validation (qRT-PCR)**			
	**Case (*n* = 3)**	**Control (*n* = 3)**	***P***	**Knee OA Cases (*n* = 54)**	**Other OA^∗^ cases (*n* = 13)**	**Control (*n* = 34)**	***P***
Age (mean ± SD)^*a*^	64.72 ± 7.81	61.73 ± 7.59	0.600	69.24 ± 9.09	62.23 ± 16.99	55.82 ± 12.37	0.049
**Gender, N (100%)**							
Male	2 (66.67)	2 (66.67)	–	13 (24.07)	3 (23.08)	18 (52.94)	0.014
Female	1 (33.33)	1 (33.33)		41 (75.93)	10 (76.92)	16 (47.06)	
**BMI**							
< 24	0	0	–	11 (20.37)	2 (15.38)	6 (17.65)	0.664
24 ≤ BMI < 28	2 (66.67)	2 (66.67)		25 (46.30)	7 (53.85)	21 (61.76)	
** ≥** 28	1 (33.33)	1 (33.33)		18 (33.33)	4 (30.77)	7 (20.59)	
**Smoking status**							
Current	0	0	0.532	9 (16.67)	2 (15.38)	12 (35.29)	0.323
Ever	1 (33.33)	2 (66.67)		10 (18.52)	3 (23.08)	6 (17.65)	
Never	2 (66.67)	1 (33.33)		35 (64.81)	8 (61.54)	16 (47.06)	
**Drinking status**							
Current	0	0	0.532	15 (27.78)	4 (30.77)	10 (29.41)	0.992
Ever	1 (33.33)	2 (66.67)		15 (27.78)	3 (23.08)	8 (23.53)	
Never	2 (66.67)	1 (33.33)		24 (44.44)	6 (46.15)	16 (47.06)	

^*a*^Median age in all subjects. *Other OA include hip, shoulder and ankle site.

### Enrichment Analysis of DEGs

**FIGURE 2:**
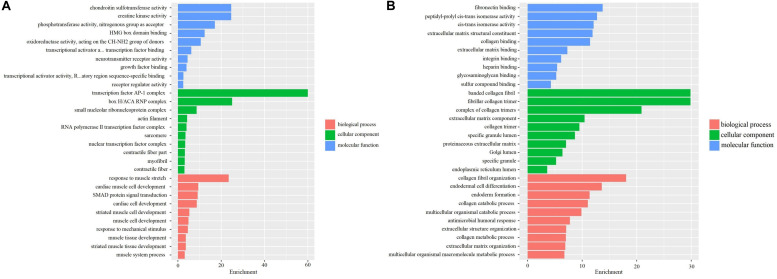
GO analyses in 3 OA cases and 3 OA-free controls in the internal samples. **(A)** GO analysis for up-regulated DEGs in 3 OA cases and 3 OA-free controls. **(B)** GO analysis for down-regulated DEGs in 3 OA cases and 3 OA-free controls.

The differently expressed mRNAs based on screening samples were performed by GO and KEGG enrichment analyses. GO analysis indicated that the up-regulated genes were primarily enriched in extracellular matrix structural constituent, glycosaminoglycan binding, and collagen binding, etc. in BP; proteinaceous extracellular matrix and extracellular matrix component, etc. in CC; extracellular structure organization and extracellular matrix organization, etc. in MF (Figure 2A). Furthermore, the down-regulated genes were mainly enriched in phosphotransferase activity, the nitrogenous group as acceptor, etc. in BP; transcription factor AP-1 complex, etc. in CC; response to muscle stretch, cardiac muscle cell development, and cardiac cell development, etc. in MF (Figure 2B). These pathways possibly participated in regulating the occurrence and progression of OA.

**FIGURE 3:**
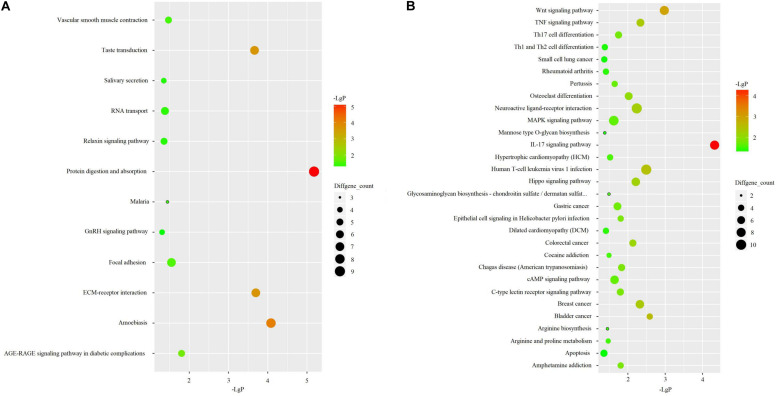
KEGG pathway analyses in 3 OA cases and 3 OA-free controls. **(A)** KEGG pathway analysis for up-regulated DEGs. **(B)** KEGG pathway analysis for up-regulated DEGs.

KEGG pathway analysis revealed that up-regulated genes were primarily enriched in pathways associated with protein digestion and absorption, amoebiasis, taste transduction, and ECM-receptor interaction (Figure 3A). Down-regulated genes were mainly associated with an IL-17 signaling pathway, wnt signaling pathway, and human T-cell leukemia virus 1 infection (Figure 3B).

### qRT-PCR Validation

To validate the expression level among the OA case group and healthy control group, 101 additional samples were collected and used for quantification, and the relative expression level was performed by qRT-PCR. The characteristics of the participants are also shown in Table 2.

**FIGURE 4:**
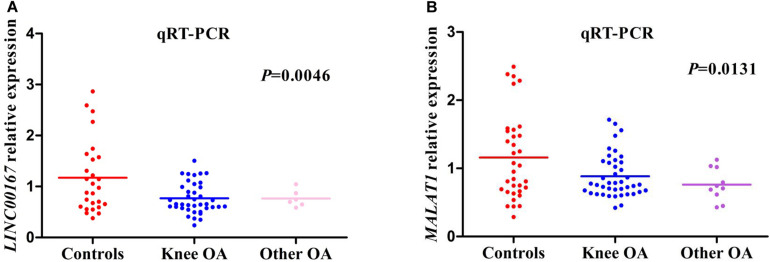
The relative expression level of hub lncRNAs in different groups. **(A)** The relative expression level of *LINC00167* in different groups. **(B)** The relative expression level of *MALAT1* in different groups.

OA cases were divided into two groups, including knee OA and other OA. Other OA groups included hip site, shoulder site, and ankle site. The relative expression level of *LINC00167* was consistent with the GSE114007 microarray data (screening stage) and presented a similar trend of down-regulation (Figure 4A). Nevertheless, the expression of *MALAT1* from the GSE114007 data (up-regulated) was inconsistent with the validation result of qRT-PCR (down-regulated) (Figure 4B). Further pairwise comparisons analysis showed that the difference of *LINC00167* and *MALAT1* between OA and non-OA was statistically significant (*P* < 0.01). There was no significant difference between knee OA and other OA groups (*P* > 0.05).

### Evaluation of Hub-lncRNA for OA Diagnosis

**FIGURE 5:**
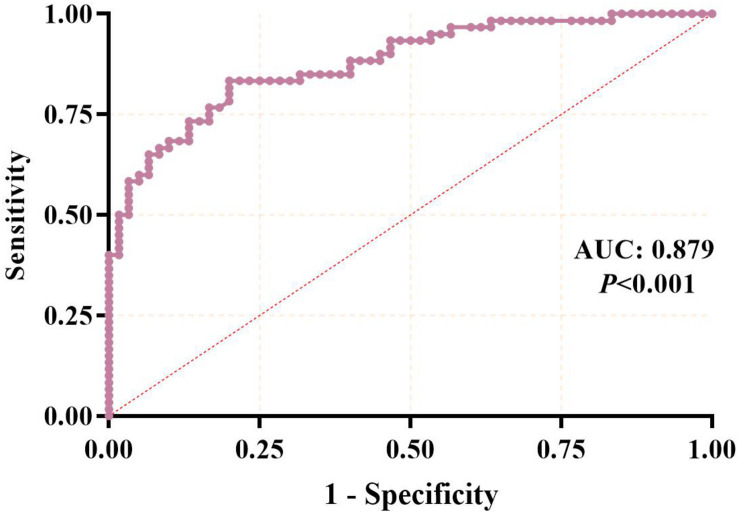
ROC curve analysis of the diagnostic value of serum lncRNA for osteoarthritis.

To investigate the potential value in OA diagnosis, the ROC curve was calculated using subjects from the third stage validation, including 60 OA cases and 60 healthy controls. In this analysis, the patients were used as true positive samples and the healthy controls were used as true negative samples. As shown in Figure 5, the AUC was 0.879, with the 95%CI of 0.819–0.938 (*P* < 0.001), indicating that a strong distinction existed between the OA cases and healthy controls and that the serum level of *LINC00167* may serve as a diagnostic biomarker for this disease.

### Association Between the Serum Levels of LINC0067 and Clinical Characteristics of OA Patients

The patients were divided into the high level group and the low level group according to the median of *LINC00167* in the serum. As displayed in Table 3, no significant associations were detected between the two groups for age, gender, BMI, and an individual’s common habits, including smoking and drinking status. Most importantly, no significant difference was found between the levels of *LINC00167* and the course of OA disease (all *P* > 0.05).

**TABLE 3:** Association between the serum levels of lncRNA00167 and clinical data for OA patients in the diagnostic test.

**Variables**	**Subgroups**	**Cases**	**High relative expression**	**Low relative expression**	***P*-value**
Gender	Male	21	11	10	0.645
	Female	39	18	21	
Age	Under 50 years	13	6	7	0.525
	Over 50 years	47	27	20	
BMI	< 24	11	4	7	0.199
	24 ≤ BMI < 28	36	22	14	
	** ≥** 28	13	5	8	
Disease course	Under 5 years	22	6	16	0.578
	Over 5 years	38	13	25	
Smoking	Current	13	7	6	0.796
	Ever	10	4	6	
	Never	37	17	20	
Drinking	Current	15	7	8	0.707
	Ever	19	11	8	
	Never	26	12	14	

## Discussion

Despite concerted efforts made in the treatment and prevention of OA, the incidence of this disease is expected to increase. It is critical to clarify the hub genes and critical signaling pathways and elucidate the pathogenesis of disease onset and progression. RNA-seq screening and independent validation studies have consistently suggested that the expression of *LINC00167* in PBL was strongly lower in OA cases than in healthy controls in a less invasive and more accessible blood sample. Our study provided obvious evidence for the replication of these screening results from RNA-seq with GEO dataset GSE114007, which may considerably enhance the stability of our findings. Taken together, *LINC00167* in PBL may be a potential diagnostic biomarker of OA. These findings need to be elucidated through further exploration and verification, but still demonstrate OA pathophysiology, providing an important step in the diagnosis of this condition and showing therapeutic promise.

In the present study, through three stages of screening and validation research, a total of 18 candidate lncRNAs were obtained and 3 hub lncRNAs were selected to confirm the reliability of the results. It was verified that *LINC00167* was down-expressed in the PBL of OA and could be detected in the early stages of OA. Our sequencing results show that the up-regulated DEGs were mainly enriched in the extracellular matrix, while the down-regulated DEGs were enriched in the *IL-17* signaling pathway and wnt signaling pathway. This suggests that these dysregulated genes and enrichment pathways may play important roles in the occurrence and progression of OA.

In our study, *LINC00167* was verified as being differently down-expressed in both human peripheral blood and the articular cartilage of OA patients. This highlights that the down-regulation of *LINC00167* is very likely involved in the pathogenesis and may be an important part of the OA process. Another previous study showed that *LINC00167* may serve as a biomarker for gastric cancer and *LINC00167* might also be associated with gastric cancer through involvement in four pathways, including “cell adhesion molecules,” “cytokine-cytokine receptor interaction,” “leukocyte transendothelial migration,” and “chemokine signaling pathway” which concern 32 genes, and the gene TNFRSF13B was highly associated with *LINC00167* (Hu et al., 2019). Importantly, TNFRSF13B is already known for its pivotal role in pain linked to inflammatory regulation, nociceptive signaling, and protein kinase functions, and differs significantly between participants with chronic pain and healthy controls (Polli et al., 2020). Additionally, *LINC00167* functioned as a sponge for microRNA miR-203a-3p, restoring the expression of the suppressor of cytokine signaling 3 (SOCS3), which further inhibited the Janus kinase (JAK)/signal transducer and activator of transcription (STAT) signaling pathway (Chen et al., 2020). Other studies have shown that enhanced SOCS3 in the human body may limit both proliferation and inflammation (Gui et al., 2017; Kong et al., 2017), demonstrating that *LINC00167* may play a protective role in OA. In addition, *MALAT1* was identified as up-regulated in the microarray data, but down-regulated in the validation of qRT-PCR. Compelling studies have shown that *MALAT1* is involved in some diseases, such as cardiovascular disease, diabetes-related complications, cancer, and metastasis, including OA (Abdulle et al., 2019; Sun and Ma, 2019; Yan et al., 2019; Zhang et al., 2019). *MALAT1* was reported to be up-regulated in OA patients and responsible for cell proliferation, apoptosis, and ECM degradation via the miR-150-5p/AKT3 axis (Zhang et al., 2019). This still requires further verification and should be explored in further studies.

To fully explore the key genes based on the previous analysis strategy, we predicted target mRNAs of the *LINC00167* by starbase online software^2^ (data are shown in Supplementary Table 1). One limitation of this study is the lack of verification of the downstream regulatory mechanisms of *LINC00167*. More investigations are warranted to better elucidate the regulatory network of *LINC00167* in patients in future studies.

This study has systematically characterized the profile of mRNAs and lncRNAs expression and their potential biological functions in blood samples between OA patients and controls. Considering the tissue specificity of lncRNAs, we focused on lncRNAs differently expressed in both cartilage (GEO dataset) and peripheral blood (RNA-seq samples) as the hub lncRNAs and further verified in an external population, which make our results more reliable. More importantly, the acquisition of blood samples is feasible as a means of early screening and diagnosis in clinical practice compared with cartilage samples.

There are still some limitations that need to be considered. Firstly, there was a small sample size involved in the RNA-seq. Further expression profiling studies with a larger sample size are still needed to validate these findings. Secondly, the difficulty of knee cartilage availability, especially donors of healthy control cartilage, limited our ability to identify and verify the different expression in OA cartilage, and normal cartilage. Thirdly, we did not collect social/economic information and medication information from patients owing to accessibility, which might influence the results. Last but not the least, although different skeletal sites (hip, shoulder, and ankle) have not been separately analyzed in the other OA groups because of limited participants, a non-significant difference was detected between the knee OA group and other OA groups. Further studies are warranted to test the veracity and credibility of this difference.

In conclusion, this study discovered that the expression of *LINC00167* in OA cases is lower than healthy controls and its expression in serum is a potentially reliable diagnostic marker for osteoarthritis patients. However, the mechanism of action remains to be further elucidated. The expression of *MALAT1* in PBL and cartilage in OA patients need to be validated in future research. Further research is warranted, to confirm the underlying biological mechanisms and illuminate feasibility as a biomarker.

## Data Availability Statement

This manuscript contains previously unpublished data. The name of the repository is GEO and accession number is GSE163552.

## Ethics Statement

The studies involving human participants were reviewed and approved by the Ethics Committee of Shanghai University of Medicine and Health Sciences. The patients/participants provided their written informed consent to participate in this study.

## Author Contributions

LJ contributed to interpretation of results and made critical revisions. YZ drafted the protocol and JS wrote the final manuscript. YC, ZM, YY, and MC participated in the data collection. QQ, XZ, and SX also made critical revisions. All authors have reviewed the final version of the manuscript and approved it for publication.

## Conflict of Interest

The authors declare that the research was conducted in the absence of any commercial or financial relationships that could be construed as a potential conflict of interest.

## Footnotes

1 http://www.ncbi.nlm.nih.gov/geo/

2 http://starbase.org

## Supplementary Material

Supplementary Figure 1 Differential expression analyses in 3 OA cases and 3 OA-free controls in the internal samples. **(A)** Volcano plot with the differential expressed mRNA. **(B)** Volcano plot with the differential expressed lncRNAs.

Supplementary Table 1 The ceRNA interaction network of *LINC00167.*

## References

[B1] Abdulle L. E. Hao J. L. Pant O. P. Liu X. F. Zhou D. D. Gao Y. (2019). MALAT1 as a diagnostic and therapeutic target in diabetes-related complications: a promising long-noncoding RNA. *Intl. J. Med. Sci.* 16 548–555. 10.7150/ijms.30097 31171906 PMC6535662

[B2] Cao L. Wang Y. Wang Q. Huang J. (2018). LncRNA FOXD2-AS1 regulates chondrocyte proliferation in osteoarthritis by acting as a sponge of miR-206 to modulate CCND1 expression. *Biomed. Pharmacother.* 106 1220–1226. 10.1016/j.biopha.2018.07.048 30119190

[B3] Cen X. Huang X. Q. Sun W. T. Liu Q. Liu J. (2017). Long noncoding RNAs: a new regulatory code in osteoarthritis. *Am. J. Transl. Res.* 9 4747–4755. 29218077 PMC5714763

[B4] Chen X. Sun R. X. Yang D. D. Jiang C. Liu Q. H. (2020). LINC00167 Regulates RPE differentiation by targeting the miR-203a-3p/SOCS3 axis. *Mol. Ther-Nucl. Acids* 19 1015–1026. 10.1016/j.omtn.2019.12.040 32044724 PMC7015824

[B5] Chu M. Zhu X. Wang C. Rong J. Wang Y. Wang S. (2017). The rs4238326 polymorphism in ALDH1A2 gene potentially associated with non-post traumatic knee osteoarthritis susceptibility: a two-stage population-based study. *Osteoarthritis Cartilage* 25 1062–1067. 10.1016/j.joca.2017.01.003 28089900

[B6] Dang X. Lian L. Wu D. (2018). The diagnostic value and pathogenetic role of lncRNA-ATB in patients with osteoarthritis. *Cell. Mol. Biol. Lett.* 23:55. 10.1186/s11658-018-0118-9 PMC6258155 30505322

[B7] Dou P. Hu R. Zhu W. Tang Q. Li D. Li H. (2017). Long non-coding RNA HOTAIR promotes expression of ADAMTS-5 in human osteoarthritic articular chondrocytes. *Pharmazie* 72 113–117. 29441864 10.1691/ph.2017.6649

[B8] Du J. Yuan Z. Ma Z. Song J. Xie X. Chen Y. (2014). KEGG-PATH: kyoto encyclopedia of genes and genomes-based pathway analysis using a path analysis model. *Mol. Biosyst.* 10 2441–2447. 10.1039/c4mb00287c 24994036

[B9] Fu M. Huang G. Zhang Z. Liu J. Zhang Z. Huang Z. (2015). Expression profile of long noncoding RNAs in cartilage from knee osteoarthritis patients. *Osteoarthritis Cartilage* 23 423–432. 10.1016/j.joca.2014.12.001 25524778

[B10] Gui T. He B. S. Gan Q. Yang C. (2017). Enhanced SOCS3 in osteoarthiritis may limit both proliferation and inflammation. *Biotech. Histochem.* 92 107–114. 10.1080/10520295.2017.1278792 28296552

[B11] Hermann W. Lambova S. Muller-Ladner U. (2018). Current treatment options for osteoarthritis. *Curr. Rheumatol. Rev.* 14 108–116. 10.2174/1573397113666170829155149 28875826

[B12] Hu Z. Yang D. Tang Y. Zhang X. Wei Z. Fu H. (2019). Five-long non-coding RNA risk score system for the effective prediction of gastric cancer patient survival. *Oncol. Lett.* 17 4474–4486. 30988816 10.3892/ol.2019.10124 PMC6447923

[B13] Huang D. W. Sherman B. T. Tan Q. Collins J. R. Alvord W. G. Roayaei J. (2007). The DAVID gene functional classification tool: a novel biological module-centric algorithm to functionally analyze large gene lists. *Genome Biol.* 8:R183. 10.1186/gb-2007-8-9-r183 PMC2375021 17784955

[B14] Huang J. Liu L. Yang J. Ding J. Xu X. (2019). lncRNA DILC is downregulated in osteoarthritis and regulates IL-6 expression in chondrocytes. *J. Cell Biochem.* 120 16019–16024. 10.1002/jcb.28880 31069838

[B15] Hunt M. A. Charlton J. M. Esculier J. F. (2019). Osteoarthritis year in review 2019: mechanics. *Osteoarthritis Cartilage* 28 267–274. 10.1016/j.joca.2019.12.003 31877382

[B16] Jarroux J. Morillon A. Pinskaya M. (2017). History, discovery, and classification of lncRNAs. *Adv. Exp. Med. Biol.* 1008 1–46. 10.1007/978-981-10-5203-3_1 28815535

[B17] Jayasuriya C. T. Hu N. Li J. Lemme N. Terek R. Ehrlich M. G. (2018). Molecular characterization of mesenchymal stem cells in human osteoarthritis cartilage reveals contribution to the OA phenotype. *Sci. Rep.* 8:7044. 10.1038/s41598-018-25395-8 PMC5935742 29728632

[B18] Kijowski R. Demehri S. Roemer F. Guermazi A. (2019). Osteoarthritis year in review 2019: imaging. *Osteoarthritis Cartilage* 28 285–295. 10.1016/j.joca.2019.11.009 31877380

[B19] Kloppenburg M. Berenbaum F. (2020). Osteoarthritis year in review 2019: epidemiology and therapy. *Osteoarthritis Cartilage* 28 242–248. 10.1016/j.joca.2020.01.002 31945457

[B20] Kong Y. Zhang Y. Zhao X. Wang G. Liu Q. (2017). Carboxymethyl-chitosan attenuates inducible nitric oxide synthase and promotes interleukin-10 production in rat chondrocytes. *Exp. Ther. Med.* 14 5641–5646. 29285104 10.3892/etm.2017.5258 PMC5740727

[B21] Kramarz B. Lovering R. C. (2019). “Gene ontology: a resource for analysis and interpretation of alzheimer’s disease data,” in *Alzheimer’s Disease*, ed. Wisniewski T. (Brisbane: Elsevier). 31895510

[B22] Langmead B. (2010). Aligning short sequencing reads with Bowtie. *Curr. Protoc. Bioinformatics.* 11:7. 10.1002/0471250953.bi1107s32 PMC3010897 21154709

[B23] Liao Y. Smyth G. K. Shi W. (2014). featureCounts: an efficient general purpose program for assigning sequence reads to genomic features. *Bioinformatics* 30 923–930. 10.1093/bioinformatics/btt656 24227677

[B24] Love M. I. Huber W. Anders S. (2014). Moderated estimation of fold change and dispersion for RNA-seq data with DESeq2. *Genome Biol.* 15:550. 10.1186/s13059-014-0550-8 PMC4302049 25516281

[B25] Maly M. R. Marriott K. A. Chopp-Hurley J. N. (2019). Osteoarthritis year in review 2019: rehabilitation and outcomes. *Osteoarthritis Cartilage* 28 249–266. 31877379 10.1016/j.joca.2019.11.008

[B26] Pearson M. J. Jones S. W. (2016). Review: long noncoding RNAs in the regulation of inflammatory pathways in rheumatoid arthritis and osteoarthritis. *Arthritis Rheumatol.* 68 2575–2583. 10.1002/art.39759 27214788 PMC5347907

[B27] Peffers M. J. Balaskas P. Smagul A. (2018). Osteoarthritis year in review 2017: genetics and epigenetics. *Osteoarthritis Cartilage* 26 304–311. 10.1016/j.joca.2017.09.009 28989115 PMC6292677

[B28] Piwowar H. Priem J. Lariviere V. Alperin J. P. Matthias L. Norlander B. (2018). The state of OA: a large-scale analysis of the prevalence and impact of open access articles. *Peer J.* 6:e4375. 10.7717/peerj.4375 29456894 PMC5815332

[B29] Polli A. Godderis L. Ghosh M. Ickmans K. Nijs J. (2020). Epigenetic and miRNA expression changes in people with pain: a systematic review. *J. Pain* 21 763–780. 10.1016/j.jpain.2019.12.002 31837447

[B30] Ramos Y. F. Bos S. D. Lakenberg N. Bohringer S. den Hollander W. J. Kloppenburg M. (2014). Genes expressed in blood link osteoarthritis with apoptotic pathways. *Ann. Rheum. Dis.* 73 1844–1853. 10.1136/annrheumdis-2013-203405 23864235

[B31] Razmara E. Bitaraf A. Yousefi H. Nguyen T. H. Garshasbi M. Cho W. C. (2019). Non-coding RNAs in cartilage development: an updated review. *Int. J. Mol. Sci.* 20:4475. 10.3390/ijms20184475 31514268 PMC6769748

[B32] Shui X. Xie Q. Chen S. Zhou C. Kong J. Wang Y. (2020). Identification and functional analysis of long non-coding RNAs in the synovial membrane of osteoarthritis patients. *Cell Biochem. Funct.* 38, 460–471. 31960487 10.1002/cbf.3491 PMC7318166

[B33] Sun Y. Ma L. (2019). New insights into long non-coding RNA MALAT1 in CAncer and metastasis. *Cancers* 11:216. 10.3390/cancers11020216 30781877 PMC6406606

[B34] van Spil W. E. Szilagyi I. A. (2019). Osteoarthritis year in review 2019: biomarkers (biochemical markers). *Osteoarthritis Cartilage* 28 296–315. 10.1016/j.joca.2019.11.007 31887390

[B35] Wu Y. Lu X. Shen B. Zeng Y. (2019). The therapeutic potential and role of miRNA, lncRNA, and circRNA in Osteoarthritis. *Curr. Gene. Ther.* 19 255–263. 10.2174/1566523219666190716092203 31333128

[B36] Xiao Y. Bao Y. Tang L. Wang L. (2019). LncRNA MIR4435-2HG is downregulated in osteoarthritis and regulates chondrocyte cell proliferation and apoptosis. *J. Orthop. Surg. Res.* 14:247. 10.1186/s13018-019-1278-7 PMC6683450 31387631

[B37] Yan Y. Song D. Song X. Song C. (2019). The role of lncRNA MALAT1 in cardiovascular disease. *IUBMB Life* 72 334–342. 10.1002/iub.2210 31856403

[B38] Zhang Y. Wang F. Chen G. He R. Yang L. (2019). LncRNA MALAT1 promotes osteoarthritis by modulating miR-150-5p/AKT3 axis. *Cell Biosci.* 9:54. 10.1186/s13578-019-0302-2 PMC6600894 31304004

[B39] Zhao Y. Xu J. (2018). Synovial fluid-derived exosomal lncRNA PCGEM1 as biomarker for the different stages of osteoarthritis. *Int. Orthop.* 42 2865–2872. 10.1007/s00264-018-4093-6 30128669

[B40] Zheng W. Lin P. Ma Y. Shao X. Chen H. Chen D. (2017). Psoralen promotes the expression of cyclin D1 in chondrocytes via the Wnt/beta-catenin signaling pathway. *Int. J. Mol. Med*. 40 1377–1384. 10.3892/ijmm.2017.3148 28949389 PMC5627873

[B41] Zhou B. F. Cooperative Meta-Analysis Group of the Working Group on Obesity in China. (2002). Predictive values of body mass index and waist circumference for risk factors of certain related diseases in Chinese adults–study on optimal cut-off points of body mass index and waist circumference in Chinese adults. *Biomed. Environ. Sci.* 15 83–96. 12046553

