# Supplementary material for: RNA Sequencing Reveals LINC00167 as a Potential Diagnosis Biomarker for Primary Osteoarthritis: A Multi-Stage Study
Source: Front Genet. 2021 Jan 14;11:539489. doi: 10.3389/fgene.2020.539489 (PMC7841430; doi:10.3389/fgene.2020.539489)
Supplement: Supplementary Figure 1 — Differential expression analyses in 3 OA cases and 3 OA-free controls in the internal samples. (A) Volcano plot with the differential expressed mRNA. (B) Volcano plot with the differential expressed lncRNAs. [file Table_1.DOCX]

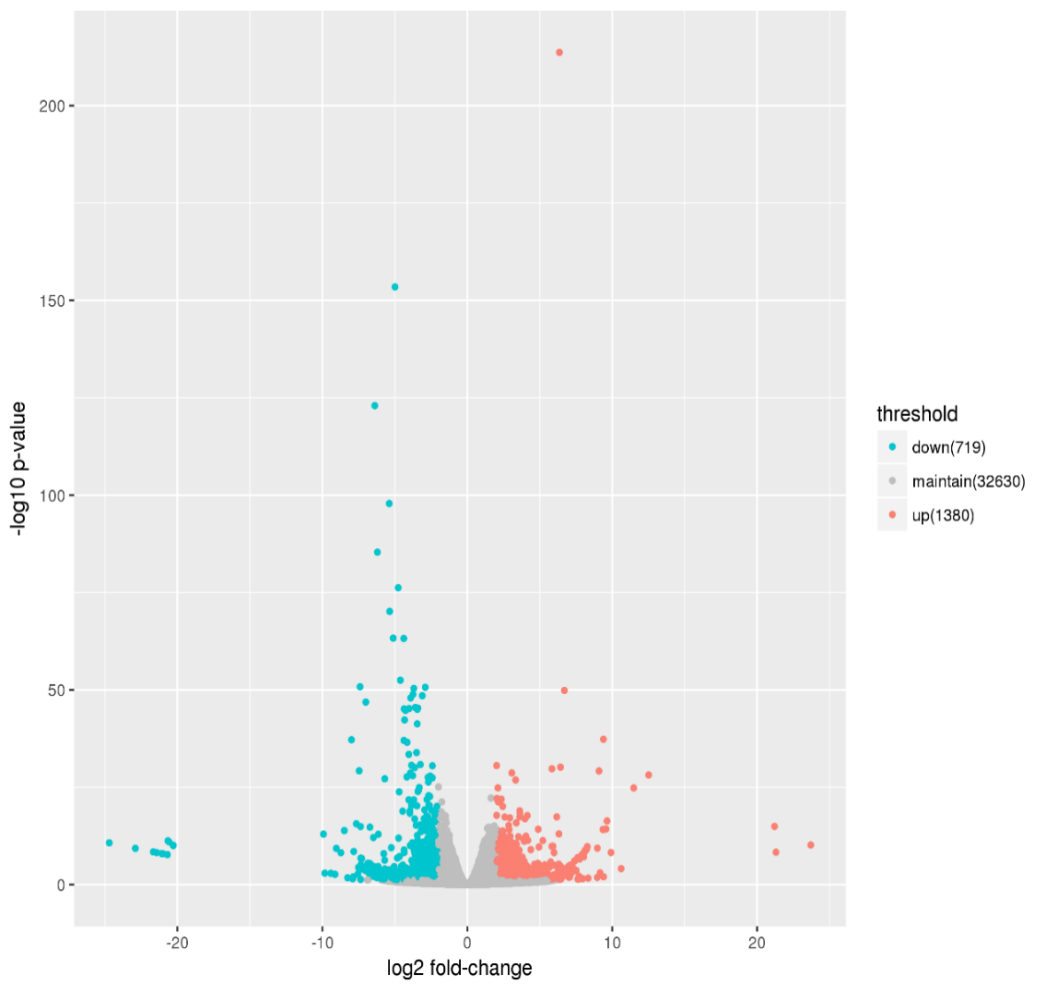

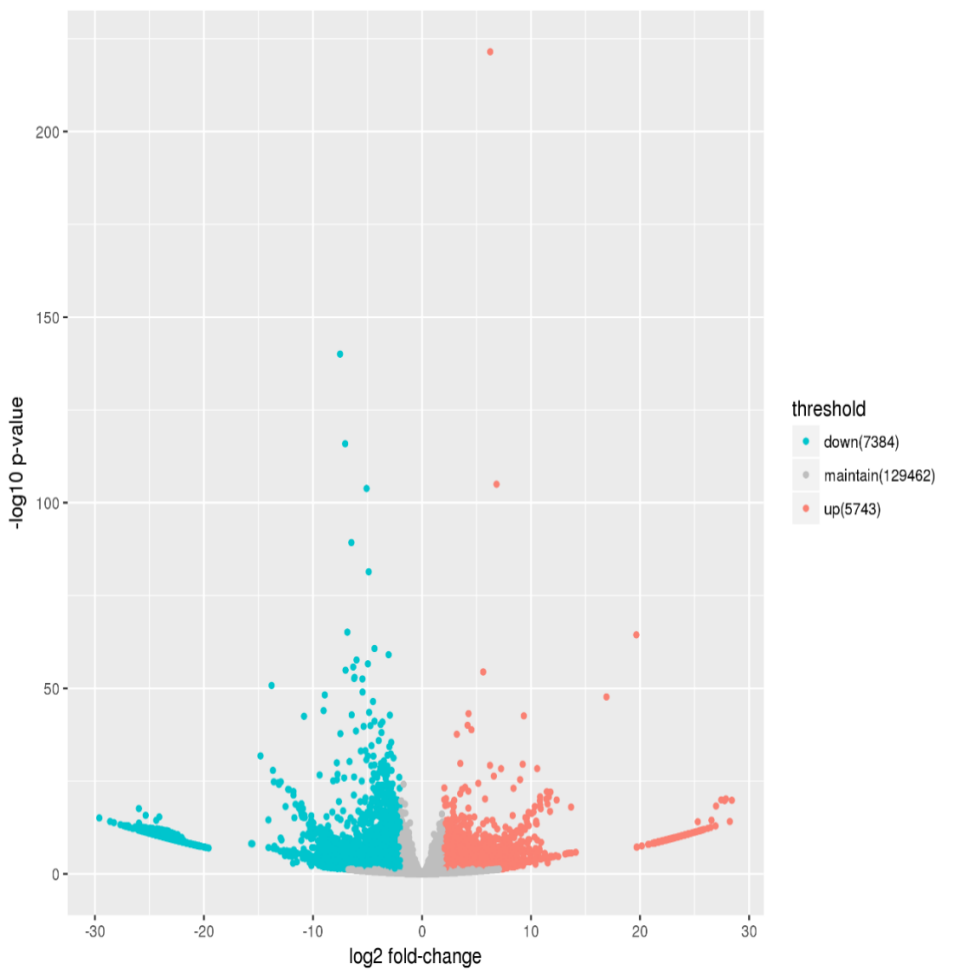


B

A

**Supplementary Figure1**. Differential expression analyses in 3 OA cases and 3 OA-free controls in the internal samples. (A) Volcano plot with the differential expressed mRNA. (B): Volcano plot with the differential expressed lncRNAs.
