# Supplementary material for: RNA Sequencing Reveals LINC00167 as a Potential Diagnosis Biomarker for Primary Osteoarthritis: A Multi-Stage Study
Source: Front Genet. 2021 Jan 14;11:539489. doi: 10.3389/fgene.2020.539489 (PMC7841430; doi:10.3389/fgene.2020.539489)
Supplement: Supplementary Table 1 — The ceRNA interaction network of LINC00167. [file Table_2.DOCX]

**Supplementary Table1**. The ceRNA interaction network of *LINC00167*

| **CeRNA gene ID** | **Name** | **Gene type** | ***P*** | **FDR** |
| --- | --- | --- | --- | --- |
| ENSG00000258611 | AC087641.1 | Processed pseudogene | 7.61E-07 | 4.26E-05 |
| ENSG00000162981 | FAM84A | Protein coding | 1.28E-06 | 4.26E-05 |
| ENSG00000130592 | LSP1 | Protein coding | 1.81E-06 | 4.26E-05 |
| ENSG00000279583 | AC009086.3 | TEC | 3.18E-06 | 4.46E-05 |
| ENSG00000268614 | AC008878.2 | Protein coding | 6.35E-06 | 7.12E-05 |
| ENSG00000090674 | MCOLN1 | Protein coding | 6.35E-06 | 7.12E-05 |
| ENSG00000133315 | MACROD1 | Protein coding | 6.35E-06 | 7.12E-05 |
| ENSG00000104825 | NFKBIB | Protein coding | 6.73E-06 | 7.12E-05 |
| ENSG00000231304 | SGO1-AS1 | Processed transcript | 1.11E-05 | 7.12E-05 |
| ENSG00000196465 | MYL6B | Protein coding | 1.11E-05 | 7.12E-05 |
| ENSG00000161544 | CYGB | Protein coding | 1.33E-05 | 7.12E-05 |
| ENSG00000204666 | AC010624.1 | Sense overlapping | 1.77E-05 | 8.27E-05 |
| ENSG00000267260 | AC020928.2 | lincRNA | 1.77E-05 | 8.27E-05 |
| ENSG00000161914 | ZNF653 | Protein coding | 1.77E-05 | 8.27E-05 |
| ENSG00000236279 | CLEC2L | Protein coding | 2.65E-05 | 9.91E-05 |
| ENSG00000259498 | TPM1-AS | antisense | 2.65E-05 | 9.91E-05 |
| ENSG00000105329 | TGFB1 | Protein coding | 2.73E-05 | 9.91E-05 |
| ENSG00000188186 | LAMTOR4 | Protein coding | 3.11E-05 | 9.91E-05 |
| ENSG00000130522 | JUND | Protein coding | 3.98E-05 | 1.17E-04 |
| ENSG00000111077 | TNS2 | Protein coding | 5.02E-05 | 1.41E-04 |
| ENSG00000197483 | ZNF628 | Protein coding | 5.19E-05 | 1.41E-04 |
| ENSG00000235821 | IFITM4P | Processed pseudogene | 6.91E-05 | 1.76E-04 |
| ENSG00000168993 | CPLX1 | Protein coding | 6.91E-05 | 1.76E-04 |
| ENSG00000125520 | SLC2A4RG | Protein coding | 6.95E-05 | 1.76E-04 |
| ENSG00000089351 | GRAMD1A | Protein coding | 1.13E-04 | 2.54E-04 |
| ENSG00000105723 | GSK3A | Protein coding | 1.24E-04 | 2.67E-04 |
| ENSG00000273413 | AL136982.7 | antisense | 2.01E-04 | 4.17E-04 |
| ENSG00000274512 | TBC1D3L | Protein coding | 2.01E-04 | 4.17E-04 |
| ENSG00000273513 | TBC1D3K | Protein coding | 2.01E-04 | 4.17E-04 |
| ENSG00000270726 | AJ271736.1 | Protein coding | 2.12E-04 | 4.17E-04 |
| ENSG00000160325 | CACFD1 | Protein coding | 2.23E-04 | 4.17E-04 |
| ENSG00000250479 | CHCHD10 | Protein coding | 2.54E-04 | 4.44E-04 |
| ENSG00000272657 | AP000317.2 | Processed transcript | 3.01E-04 | 5.10E-04 |
| ENSG00000102996 | MMP15 | Protein coding | 3.46E-04 | 5.70E-04 |
| ENSG00000253982 | AC100810.1 | Antisense | 3.53E-04 | 5.70E-04 |
| ENSG00000233913 | RPL10P9 | Processed-pseudogene | 3.53E-04 | 5.70E-04 |
| ENSG00000172137 | CALB2 | Protein coding | 3.71E-04 | 5.70E-04 |
| ENSG00000275620 | AL121827.2 | Antisense | 4.01E-04 | 5.91E-04 |
| ENSG00000227057 | WDR46 | Protein coding | 4.01E-04 | 5.91E-04 |
| ENSG00000267261 | AC099811.2 | Protein coding | 4.75E-04 | 6.65E-04 |
| ENSG00000172830 | SSH3 | Protein coding | 4.83E-04 | 6.65E-04 |
| ENSG00000079313 | REXO1 | Protein coding | 5.48E-04 | 7.31E-04 |
| ENSG00000242262 | AC092597.1 | Processed-pseudogene | 6.22E-04 | 8.10E-04 |
| ENSG00000103326 | CAPN15 | Protein coding | 6.57E-04 | 8.37E-04 |
| ENSG00000259112 | NDUFC2-KCTD14 | Protein coding | 6.66E-04 | 8.37E-04 |
| ENSG00000279812 | AC120057.4 | TEC | 6.66E-04 | 8.37E-04 |
| ENSG00000143862 | ARL8A | Protein coding | 8.75E-04 | 1.04E-03 |
| ENSG00000234432 | AC092171.3 | LincRNA | 1.00E-03 | 1.17E-03 |
| ENSG00000260417 | AC092127.1 | LincRNA | 1.08E-03 | 1.24E-03 |
| ENSG00000273420 | AC008738.7 | 3 prime overlapping ncRNA | 1.11E-03 | 1.25E-03 |
| ENSG00000243766 | HOTTIP | Antisense | 1.39E-03 | 1.53E-03 |
| ENSG00000263069 | AC124319.2 | Antisense | 1.39E-03 | 1.53E-03 |
| ENSG00000262096 | PCDHB19P | Unprocessed pseudogene | 1.39E-03 | 1.53E-03 |
| ENSG00000253729 | PRKDC | Protein coding | 1.54E-03 | 1.60E-03 |
| ENSG00000249962 | AL157400.5 | Processed pseudogene | 1.85E-03 | 1.88E-03 |
| ENSG00000261221 | ZNF865 | Protein coding | 1.85E-03 | 1.88E-03 |
